# PAK1 activation drives divergent resistance mechanisms to aromatase inhibition and tamoxifen in a luminal: A breast cancer model

**DOI:** 10.1002/1878-0261.70286

**Published:** 2026-06-05

**Authors:** Luisa Schwarzmüller, Janina Müller, Efstathios‐Iason Vlachavas, Lukas Beumers, Lena Fleischhacker, Sara Burmester, Angelika Wörner, Sabine Karolus, Dominic Helm, Cindy Körner, Stefan Wiemann

**Affiliations:** ^1^ Division of Molecular Genome Analysis German Cancer Research Center (DKFZ) Heidelberg Germany; ^2^ Faculty of Biosciences University of Heidelberg Heidelberg Germany; ^3^ Proteomics Core Facility German Cancer Research Center (DKFZ) Heidelberg Germany

**Keywords:** endocrine therapy resistance, ER‐positive breast cancer, PAK1, phosphoproteomics

## Abstract

Breast cancer is the most frequent malignancy and the second leading cause of cancer‐related mortality in women. Estrogen receptor‐positive (ER+) tumors are treated with endocrine therapies such as tamoxifen or aromatase inhibitors (AI), aimed at disrupting estrogen signaling. While these therapies are initially effective, resident tumor cells can develop resistance, leading to relapse. The p21‐activated kinase 1 (PAK1), a regulator of oncogenic signaling pathways, has been implicated in tamoxifen resistance. However, it remains unclear whether PAK1 also affects the response to other endocrine therapies. Here we show PAK1 activity was elevated in tamoxifen‐resistant and long‐term estrogen‐deprived MCF7 cell lines and showed enhanced responsiveness to EGF stimulation. Inhibition of PAK1 effectively reduced cell proliferation in both models, with distinct effects on PAK1 downstream substrates. In the tamoxifen resistance context, PAK1 inhibition induced activation of the pro‐apoptotic protein BAD and triggered apoptosis while proliferation‐related kinases were suppressed in the estrogen‐deprived model. Our findings position PAK1 as a mediator of resistance to endocrine therapies suggesting that targeting PAK1 may present a novel strategy to overcome endocrine therapy resistance in ER+ breast cancer.

## Graphical abstract

Breast cancer remains a major cause of cancer death in women, frequently developing endocrine therapy resistance. This study demonstrates that upregulated p21‐activated kinase 1 (PAK1) activity drives resistance to tamoxifen and long‐term estrogen deprivation in ER+ breast cancer models. PAK1 inhibition abrogates proliferation and induces apoptosis, establishing PAK1 as a potential therapeutic target to circumvent endocrine resistance.

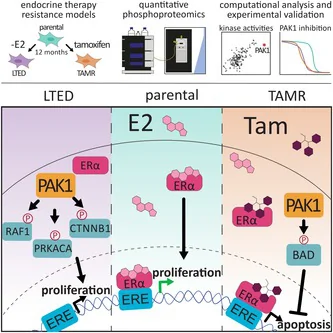

**Abbreviations**
AI: aromatase inhibitors
DIA: data‐independent acquisition
ER+: estrogen receptor‐positive
LTED: long‐term estrogen deprived
MS: mass spectrometry
TAMR: tamoxifen‐resistant

## Introduction

Breast cancer has remained the most frequent malignancy and the second leading cause of cancer‐related death in women worldwide [1, 2]. As breast cancer comprises a heterogeneous group of diseases, molecular subtyping of tumors is commonly performed in the clinics. This is mostly based on the assessment of estrogen receptor (ER), progesterone receptor (PR), human epidermal growth factor receptor 2 (HER2), and Ki‐67 expression [3]. Around two‐thirds of breast tumors are classified as ER+ [4], and the respective patients are treated with endocrine therapies to interfere with estrogen signaling [4, 5]. Tamoxifen, a modulator of receptor activation, and aromatase inhibitors depriving tumors of estrogen, are first‐line therapies in pre‐ and postmenopausal patients, respectively [5]. Despite the initial effectiveness of endocrine therapies, up to 40% of patients presenting with late‐stage disease at diagnosis ultimately develop resistance, relapse, and have a mostly poor prognosis [6]. Resistance to endocrine therapies can be mediated by various mechanisms, including mutations in *ESR*1 [7], the gene encoding ERα, upregulation of receptor tyrosine kinases, notably EGFR and HER2, and activation of downstream MAPK and PI3K pathways [8, 9, 10, 11, 12, 13, 14]. Several of these resistance mechanisms of endocrine resistance have been uncovered with the help of therapy‐resistant cell line models, which are commonly generated by long‐term cultivation of cancer cell lines under therapeutic selective pressure [15]. Along these lines, we have previously described the upregulation of EGFR and HER2 signaling in two MCF7 cell line models of endocrine resistance [16]. These models had been generated by long‐term cultivation of MCF7 cells in the presence of tamoxifen (TAMR) or in media deprived of estrogen (LTED), the latter being a model for clinical aromatase inhibition. Similar TAMR and LTED models have been established from a number of breast cancer cell lines and demonstrate highly heterogeneous and likely context‐specific adaptations compared to their respective parental cells, as summarized by the EstroGene2.0 database [17].

PAK1, the p21‐activated kinase 1, has been suggested as one possible player in endocrine therapy resistance, specifically in the context of tamoxifen treatment [18, 19]. PAK1 is a central node that integrates signals from CDC42/RAC1, PI3K/AKT, EGFR/HER2/MAPK, and Wnt/β‐Catenin pathways to regulate diverse cellular functions, including cytoskeletal organization, cell growth, survival, and migration [20, 21, 22]. Activated PAK1 signaling has been implicated in multiple cancer types and several small molecule inhibitors have been developed [23, 24]. In breast cancer, the *PAK1* gene is frequently amplified, especially in the ER+ subtype [25, 26]. Holm *et al*. found high expression of *PAK1* in 19% of premenopausal ER+ breast cancer patients. This was strongly correlated with higher malignancy as well as reduced recurrence‐free survival upon tamoxifen therapy [19]. Gonzalez et al. showed that *PAK1* phosphorylation increased two‐fold in response to tamoxifen treatment already after 24 h [18]. Additionally, they showed that in tamoxifen‐resistant MCF7, inhibition of the RAC1‐PAK1 signaling axis using a RAC1‐inhibitor restored the antiproliferative effects of tamoxifen. While the role of PAK1 in therapy resistance to tamoxifen thus seems to be established, it remains unknown whether PAK1 might have a causal relation also with resistance to estrogen deprivation via inhibition of aromatase. Such activity might prioritize inhibition of PAK1 as a potential clinical strategy to treat endocrine therapy‐resistant tumors in postmenopausal patients.

Since cellular signaling processes are highly dynamic and mainly regulated at the protein level via post‐translational mechanisms, such as phosphorylation [27], extending molecular analyses beyond genomic and transcriptomic data is crucial to assess the functional makeup of cancer cells. Here, we employed global, quantitative mass spectrometry (MS)‐based total proteomics and phosphoproteomics to gain a detailed understanding of two endocrine therapy resistance models, MCF7 long‐term estrogen deprived (LTED) and TAMR, representing resistance to aromatase inhibition and tamoxifen, respectively. The use of global phosphoproteomics data allowed us to assess the activities of kinases, which are frequently the drivers of cancer and are prime targets of therapeutic drugs [28]. In time‐resolved phosphoproteomics experiments, we observed PAK1 as one of the most active kinases in both LTED and TAMR compared to parental cells and saw further and prolonged PAK1 activation upon EGF stimulation. Both resistance models displayed similar dependence on PAK1 for proliferation. Pharmacologic inhibition of PAK1 identified both shared and distinct effects on PAK1 targets in LTED and TAMR conditions. While PAK1 drives proliferation in LTED, it rather inhibits apoptosis in TAMR. Our analyses revealed that PAK1 plays a central role in both LTED and TAMR resistance models and, via regulation of different downstream targets, might still be a common target to interfere with endocrine therapy resistance.

## Materials and methods

### Cultivation of cell lines

MCF7 (Cellosaurus CVCL_0031) cell lines were a kind gift from Prof. Luca Magnani (Imperial College London) [15]. Cell lines were authenticated using STR profiling and regularly tested for potential mycoplasma contamination. Cell lines were cultivated in Dulbecco's modified Eagle medium (DMEM) supplemented with 10% FCS, 50 units/mL penicillin, 50 μg/mL streptomycin sulfate (Invitrogen AG, Carlsbad, CA, USA), and 10–8 m 17‐β‐estradiol (E2, Sigma‐Aldrich, Saint‐Louis, MI, USA) at 37 °C with 5% CO2 in a humidified incubator. Tamoxifen‐resistant and long‐term estrogen‐deprived cells were either maintained in the presence of 100 nm of the active metabolite of tamoxifen, 4‐hydroxytamoxifen (4‐OHT, Sigma‐Aldrich), or cultured in estrogen‐free DMEM (w/o phenol red) supplemented with 10% charcoal‐stripped FCS, respectively.

### Inhibitor treatment and proliferation assay

Cells were plated in black clear‐bottom 96‐well plates (Greiner Bio‐One International GmbH, Kremsmünster, Austria) and treatment with the PAK1 inhibitor NVS‐PAK1‐1 (Cayman Chemical, MI, USA) resuspended in dimethyl‐sulfoxide (DMSO, Sigma‐Aldrich) for seven days. Cells were stained with the Hoechst‐33 342, PI, or Annexin‐V FITC (Thermo Fisher Scientific) and imaged using the IXM C microscope (Molecular Devices, San Jose, CA, USA). Images were analyzed using the Multi‐wavelength Cell Scoring module integrated in the MetaXpress Software (Molecular Devices, San Jose, CA, USA). Specifically, cells were detected based on Hoechst‐33 342 signal and scored for costaining with PI and Annexin‐V FITC based on their fluorescence intensity above local background. Proliferation, cell death, and apoptosis were determined relative to DMSO control.

### EGF stimulation

Cells were starved with FCS‐depleted media for 24 h. The following day, cells were stimulated with 5 nm epidermal growth factor (EGF, Sigma‐Aldrich, #E9644), and samples were collected at the indicated time points. Following two washing steps with PBS, cells were lysed in RIPA buffer (Thermo Fisher Scientific) that was supplemented with 10 mm NaF, 1 mm Na_3_VO_4_, cOmplete EDTA‐free protease inhibitor (Merck), PhosSTOP phosphatase inhibitor (Merck), 250 U/mL Benzonase (Merck), and 10 U/mL DNase (Qiagen). Lysates were kept on ice for 30 min and then centrifuged for 30 min at 15 000 × ***g*** and 4 °C. Supernatants were then transferred to fresh tubes. Protein concentrations were determined using the Pierce BCA assay (Thermo Fisher Scientific), and lysates were stored at −80°C until further use.

### Sample preparation workflow for phosphoproteomics

Five hundred μg of protein were precipitated according to Wessel and Flügge [29], protein pellets were dried for 15 min at room temperature and stored at −20 °C. For tryptic digestion, protein pellets were dissolved in 8 m urea, supplemented with 100 mm NaCl, 50 mm TEAB, cOmplete EDTA‐free protease inhibitor (Merck) and PhosSTOP phosphatase inhibitor (Merck). Disulfide bridges were reduced with 10 mm DTT for 1 h at 27 °C, then alkylated with 30 mm IAA for 30 min at room temperature. Reactions were quenched with 10 mm DTT and incubated for 15 min at room temperature. Lysyl endopeptidase (LysC, Fujifilm) was added in a 1 : 100 enzyme‐to‐protein ratio, and proteins were digested for 4 h at 30 °C. Then, the urea concentration was diluted to 1.6 m, Trypsin was added in a 1 : 50 ratio and the digestion continued for 16 h at 37 °C. Formic acid (FA) was added to a final concentration of 2% (v/v) to terminate digestion. Peptides were desalted using Sep‐Pak C18 cartridges (Waters). The cartridges were conditioned with 100% acetonitrile, washed with 80% acetonitrile containing 0.6% acetic acid and equilibrated twice with 2.5% formic acid. Peptide solutions were loaded, and the flow‐through was collected and loaded a second time. Bound peptides were washed four times with 2.5% formic acid and then eluted twice with 80% acetonitrile containing 0.6% acetic acid. The eluate was vacuum centrifuged to dryness and stored at −20 °C.

For enrichment of phosphorylated peptides, an Immobilized Metal Affinity Chromatography (IMAC) column (Thermo Fisher Scientific) was charged with 25 mm iron chloride (FeCl_3_) in 100 mm acetic acid at a flow rate of 0.2 mL/min for 30 min. Subsequently, the column was rinsed with 0.1% formic acid at a flow rate of 4 mL/min for 4 h. Dried peptides were reconstituted in 30% acetonitrile with 0.07% TFA and were then loaded onto the IMAC column with a flow rate of 0.2 mL/min. Elution of phosphorylated peptides was achieved with a gradient of ammonium (NH_4_OH). The phospho fraction was collected and vacuum centrifuged to dryness.

Finally, the enriched phosphopeptides were desalted by using Stop and Go Extraction (STAGE) tips. Briefly, three layers of C18 material (Supelco) were placed in a pipette tip, prewetted with methanol, washed with 80% acetonitrile in 0.1% trifluoroacetic acid (TFA), and equilibrated with 0.1% TFA. The dried peptides were reconstituted in 0.1% TFA and loaded onto the STAGE tip. Subsequently, the peptides were washed with 0.1% TFA, followed by two elution steps with 60% and 80% acetonitrile, respectively. The eluate was vacuum centrifuged to dryness and stored at −20°C until further analysis.

For the MS analysis of the PAK1 inhibitor‐treated samples, protein clean‐up was performed with 55 μg protein material as input following the automated single‐pot solid phase enhanced sample preparation (SP3) workflow [30] adapted by Müller et al. [31] on an Agilent Bravo liquid handling robot. Proteins were digested with Trypsin in a protease‐to‐protein ratio of 1 : 25 for 16 h at 37 °C. Peptides were vacuum centrifuged to dryness and subsequently enriched for phosphorylation on the Agilent Bravo liquid handling robot following the Agilent application note (https://www.agilent.com/cs/library/applications/5991-6073EN.pdf).

### Liquid chromatography

For LC–MS/MS analysis, peptides were dissolved in ULC/MS grade water containing 0.1% trifluoracetic acid (TFA) and 2.5% 1,1,1,3,3,3‐Hexafluoro‐2‐propanol (HFIP), followed by sonication for 5 min. For phospho‐enriched samples, the reconstitution buffer additionally contained 50 mm citric acid. The samples were transferred to autosampler vials and placed in the autosampler module of the Ultimate 3000 (Thermo Fisher Scientific) liquid chromatography (LC) system. The LC was operated at a flow of 300 nL/min, and the column was heated to 35 °C. Peptides were first loaded onto a trapping column (Thermo Fisher Scientific, 160 454) in the presence of 98% loading buffer A (0.1% TFA in water) and 2% loading buffer B (0.1% TFA in acetonitrile). Peptides were then transferred to the analytical column (Waters, 186 008 795, BEH C18 130 Å 1.7 μm 75 × 250 mm), from which they were separated by hydrophobicity with a linear gradient of 4%–30% acetonitrile. For phosphopeptide‐enriched samples, a gradient of 2%–28% acetonitrile was applied. Separated peptides were ionized by electrospray ionization (ESI) with an applied voltage of 2200 V.

### Mass spectrometry measurement

The Orbitrap Exploris 480 mass spectrometer (Thermo Fisher Scientific) was operated in DIA mode. MS1 scans were acquired at a resolution of 120 K and covered the range from 350 to 1400 m/z. Maximum injection time was 45 ms and the automated gain control (AGC) target was set to 3e6. MS2 scans were acquired in 47 precursor isolation windows of variable width and 1 m/z overlap that covered the range from 400 to 1000 m/z (up to 1200 m/z for phospho‐enriched samples). The orbitrap was operated at a resolution of 30 K, and a normalized collision energy of 28% (26% for phospho‐enriched samples) was applied. Maximum injection time was 54 ms, and the automated gain control (AGC) target was set to 1e6. The total cycle time of the method equaled 3.6 s.

### MS raw data search

Peptide and protein identification and quantification from DIA raw data was performed with Spectronaut (Biognosys, version 17) in library‐free (directDIA+) mode. For phosphopeptides, the newly developed identification and localization algorithm implemented in Spectronaut was used [32]. Identified phosphorylated peptides were site‐collapsed using the Perseus [33] (version 1.6.2.3) plug‐in Peptide Collapse, as described by Bekker‐Jensen et al. [32], with a localization cut‐off at 0.95.

### Data preprocessing and differential expression analysis

Proteomic data were analyzed using R [34] (v. 4.0.2) implemented in RStudio [35] (v. 1.3.1093). Protein or phosphosite intensities were normalized using the R packages vsn [36] (v. 3.70.0) and SummarizedExperiment (v. 1.32.0), accordingly. Moreover, we adapted a filtering method available in the PhosR package [37] (v. 1.4.0), to keep proteins or phosphosites that are present in at least 2 out of 3 replicate samples in at least one condition. Differential protein expression analysis for the respective comparisons was performed using the limma [38] R package (v. 3.50.3). Resulting *P*‐values were adjusted for multiple hypothesis testing using Benjamini–Hochberg method [39].

### Inference of differentially activated kinases

Relative kinase activities were determined based on the T‐statistics of the differential phosphosite abundance analysis. The OmnipathR [40] package (v. 3.2.8) was used to retrieve kinase–substrate relationships. To ensure the reliability of our approach, we excluded interactions that were solely present in ProtMapper [41] and not corroborated by any other source. Subsequently, the decoupleR [42] package (v. 2.1.6) was employed to estimate the relative activity scores by weighting the molecular readouts of its targets according to their mode of regulation (activation or inhibition) and their relative likelihood. Only kinases with at least 5 phosphosite targets present in the analyzed phosphoproteomic data were considered for this analysis.

### Gene set overrepresentation analysis

Proteins with an adjusted *P*‐value < 0.05 and a fold change greater than 2 or smaller than 0.5, were defined as up‐ or downregulated proteins, respectively. Overrepresentation analysis of Reactome pathways in these lists of significant proteins was performed using the compareCluster function of the clusterProfiler R package (v. 4.10.1).

### Two‐way ANOVA

Two‐way ANOVA was performed on the filtered and normalized phosphoproteome data using limma (v. 3.58.1) with a design that includes the cell line (MCF7 parental, LTED, or TAMR) and the time point of EGF stimulation (design = model.matrix(~cell_line+time)). This design was fit to the phosphoproteome data set using the lmFit function, and statistics were performed using the eBayes function. *P*‐values were adjusted for multiple hypothesis testing with the Benjamini‐Hochberg method. Reactome gene set enrichment analysis was performed on the proteins that were found to be significantly differentially phosphorylated using the enrichPathway function of the ReactomePA package (v. 1.46.0). To identify overrepresented kinases that are responsible for the phosphorylation of these ANOVA‐significant sites, hypergeometric testing was performed using the phyper function of the stats R package (v. 4.3.1).

### Gene expression analysis by RT‐qPCR

MCF7 LTED cells were treated with 6 μM NVS‐PAK1‐1 for 60 min or DMSO control (*n* = 3). Subsequently, the culture medium was exchanged for either LTED medium (without estrogen) or full growth medium (with estrogen), and the cells were incubated for 24 h. Then, cells were lysed, mRNA was extracted, and the expression of *PAK1, five* estrogen response genes, and *ACTB* and *PUM1* as housekeeping genes was measured by RT‐qPCR (primer sequences in Table 1) and analyzed using the ddCt method normalized to cells without NVS‐PAK1‐1 in LTED medium without estrogen.

**Table 1:** List of primers used in RT‐qPCR.

Target gene	Forward primer	Reverse primer
*PAK1*	CCTCTGCCTCCAAACCCAG	CTGCTCTGGCATTCCCGTAA
*MYC*	CACCAGCAGCGACTCTGA	GATCCAGACTCTGACCTTTTGC
*MYB*	TTTCAGTCATCTGTTCCATT	CACTTGGGGAAAACAAGGTG
*CD44*	GACACCATGGACAAGTTTTGG	CGGCAGGTTATATTCAAATCG
*BCL2*	TACCTGAACCGGCACCTG	GCCGTACAGTTCCACAAAGG
*CCND1*	GAAGATCGTCGCCACCTG	GACCTCCTCCTCGCACTTCT
*ACTB*	CATGTACGTTGCTATCCAGGC	CTCCTTAATGTCACGCACGAT
*PUM1*	TCACATGGATCCTCTTCAAGC	CCTGGAGCAGCAGAGATGTT

### Data visualization

Used functions that are not contained in base R: dotplot (clusterProfiler, v. 4.10.1), pheatmap (pheatmap, v. 1.0.12), textplot (wordcloud, v. 2.6), fviz_pca_ind (factoextra, v. 1.0.7).

For illustrations, Adobe Illustrator 2024 and Inkscape (v.1.4.2) were used.

## Results

### PAK1 is strongly overexpressed and activated in the MCF7 LTED cell line model

To study novel mechanisms underlying endocrine therapy resistance in an isogenic background, we utilized two endocrine therapy‐resistant cell line models that have been derived from the luminal A breast cancer cell line MCF7. The parental cell line had been cultivated either in the presence of tamoxifen or deprived of estrogen for 12 months, resulting in tamoxifen‐resistant (TAMR) and long‐term estrogen‐deprived (LTED) cell lines, respectively [15, 16, 43, 44]. The latter condition mimics resistance to therapeutic inhibition of aromatase.

Using these MCF7 LTED and TAMR models as well as the corresponding parental cells, we performed unbiased total proteome (Data S1) and phosphoproteome (Data S2) analysis employing data‐independent acquisition mass spectrometry (DIA MS) to assess changes in protein levels and in pathway and kinase activities upon acquisition of endocrine therapy resistance. Total proteome analysis identified a total of 7876 protein groups with at least 95% overlap between biological replicates (Fig. S1A,B). Principal component analysis suggested distinct adaptations in the two resistant cell line models (Fig. 1A), and Reactome pathway overrepresentation analysis revealed several uniquely deregulated pathways, such as upregulated cholesterol biosynthesis in LTED and higher abundance of proteins involved in gluconeogenesis in TAMR, suggesting treatment‐specific metabolic alterations (Figs 1B and S1C,D; Data S3, S4). In line with our proteomic findings, increased cholesterol synthesis has previously been implicated with endocrine therapy resistance specifically in the LTED condition by Nguyen et al. who reported that 27‐hydroxycholesterol, a downstream metabolite of cholesterol, can activate ERα and thus compensate for the lack of estrogen [15]. A preclinical study has demonstrated that combining tamoxifen treatment with cholesterol depletion (acetyl plumbagin) reduced cell proliferation in MCF7 and MCF7‐LTED cells [45].

**Fig. 1:**
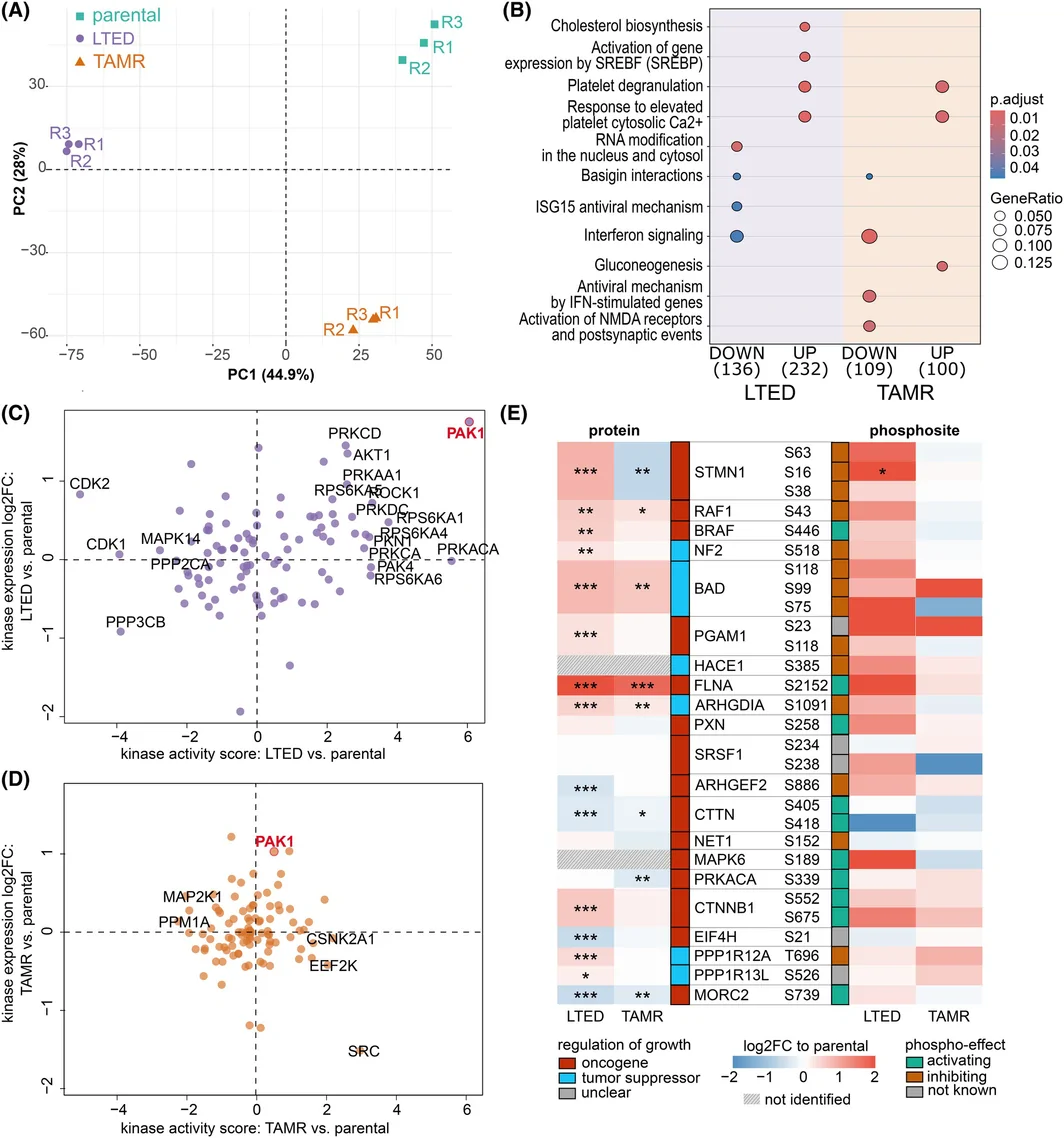
Proteomic and phosphoproteomic changes in MCF7 long‐term estrogen deprived (LTED) and tamoxifen‐resistant (TAMR) cell lines compared to parental cells. Total proteomic and phosphoproteome fractions of MCF7 parental, LTED and TAMR cells (500 μg protein input for SP3 clean‐up, with phosphopeptide enrichment for phosphoproteome analysis) were analyzed by DIA LC–MS/MS (*n* = 3). Peptides and proteins were identified and quantified using Spectronaut (v17). Phosphorylated peptides were collapsed to the site‐level using the Perseus plug‐in PeptideCollapse. (A) Principal component analysis of the total proteome analysis for parental, LTED, and TAMR conditions. R1‐3 = replicates 1–3. (B) Statistical analysis was performed using the eBayes function of the limma R package. Reactome pathway overrepresentation analysis was performed, using a threshold of fold change >2 (UP) or <0.5 (DOWN) and an adjusted *P*‐value < 0.05 in a comparison of LTED and TAMR vs. parental cells, respectively. (C,D) Statistical comparison was performed on the identified, localized, and quantified phosphosites. Kinase activities were calculated based on the t‐statistics of the respective comparison, using decoupleR and Omnipath [87]. Kinase activities and fold changes of protein abundance levels compared to parental MCF7 cells were plotted for LTED (C) and TAMR (D). Kinases with activity scores greater than 2 or below −2 are highlighted. PAK1 is additionally highlighted in TAMR. (E) Abundance of PAK1 target proteins and phosphorylation levels of known PAK1 substrate sites on these proteins [40] are shown as log2 fold change compared to parental. Significant *P*‐values after BH‐adjustment are highlighted: *** = *P*‐value < 0.001, ** = *P*‐value < 0.01, * = *P*‐value < 0.05.

Signaling pathways are not only regulated at the level of protein abundance but also by post‐translational modifications, mostly phosphorylation, of central proteins [27]. To reveal potential mechanisms and druggable drivers of therapy resistance, we utilized the quantitative phosphoproteome data in combination with the Omnipath [40] database of known kinase–substrate relationships to determine kinase activities [40]. These were calculated based on T‐statistics, in comparison to the parental cells, of the intensities in phosphosite substrates annotated for each kinase (Data S5 and S6) [40]. Here, PAK1 stood out as the most significantly activated kinase and was also the protein with the highest upregulated protein expression level in the LTED cells (Fig. 1C). In contrast, PAK1 activity was not increased in TAMR cells (Fig. 1D), while expression was upregulated also there. A similar apparent disparity between protein levels and activities has been described before and has been associated with various mechanisms, such as post‐translational modifications, cofactor requirements, and subcellular localization [46, 47]. Our findings thus support the importance of assessing kinase activities rather than relying solely on protein abundance.

PAK1 has already been described as a driver in acquired tamoxifen resistance via inhibition of RAC1 [18, 19], and, in line with our findings, its resistance driver activity has been associated with strong overexpression of the gene [19, 20, 25]. As PAK1‐activity was increased only in the LTED cells, we investigated the specific functions PAK1 might have in the LTED context. There, the abundance of known downstream PAK1 target proteins with established oncogenic activities was upregulated, such as RAF1 [48], BRAF [49], CTNNB1 [50], and FLNA [51] (Fig. 1E). Next, we inspected the phosphorylation status of these proteins at their respective PAK1 substrate sites, as annotated in the Omnipath database. This analysis confirmed preferential phosphorylation of activating marks in PAK1 target proteins encoded by oncogenes (e.g., BRAF, FLNA, CTNNB1) and inactivating marks in tumor suppressor genes (e.g., NF2, BAD, PPP1R12A) (Fig. 1E).

In conclusion, total proteome and phosphoproteome analysis of the two resistance models revealed context‐specific adaptations compared to the parental MCF7, for example, in proteins involved in cholesterol biosynthesis or gluconeogenesis in LTED and TAMR, respectively. Combined, the total proteomic and phosphoproteomic data suggested that PAK1 might be a candidate kinase contributing to therapy resistance, also in the LTED context.

### Both resistance models are more sensitive to PAK1 inhibition than the parental cells

As PAK1 is a known driver in acquired tamoxifen resistance [15, 16, 52] and since we had seen high levels and strong activation of PAK1 particularly in our MCF7 LTED model, we hypothesized that PAK1 might be involved also in resistance to estrogen deprivation by aromatase inhibitors. To test whether PAK1 activity is relevant for proliferation and survival in our resistance models, we treated the parental, LTED, and TAMR cell lines with different doses of PAK1‐specific small molecule inhibitor NVS‐PAK1‐1 [53] and quantified the effects on cell proliferation. Indeed, the LTED (IC50: 5.4 μM) and the TAMR (IC50: 7.2 μM) cell lines were significantly more sensitive to PAK1 inhibition than the parental cells (IC50: 15.7 μM, Fig. 2A), after seven days of treatment. This suggests that proliferation of both endocrine therapy resistance models relies on active PAK1 signaling. We thus hypothesized that the dependence on PAK1 might be a common mechanism beyond tamoxifen, to compensate for the loss of estrogen‐stimulated signaling. To test this for the LTED model, we next re‐introduced estrogen into the culture medium of LTED cells and measured *PAK1* mRNA levels after 4, 8, and 12 weeks. Upon re‐exposure to estrogen, *PAK1* expression strongly decreased in the LTED cells and remained low for the entire duration of the experiment (Fig. 2B), suggesting a direct relation between estrogen availability and PAK1 levels.

**Fig. 2:**
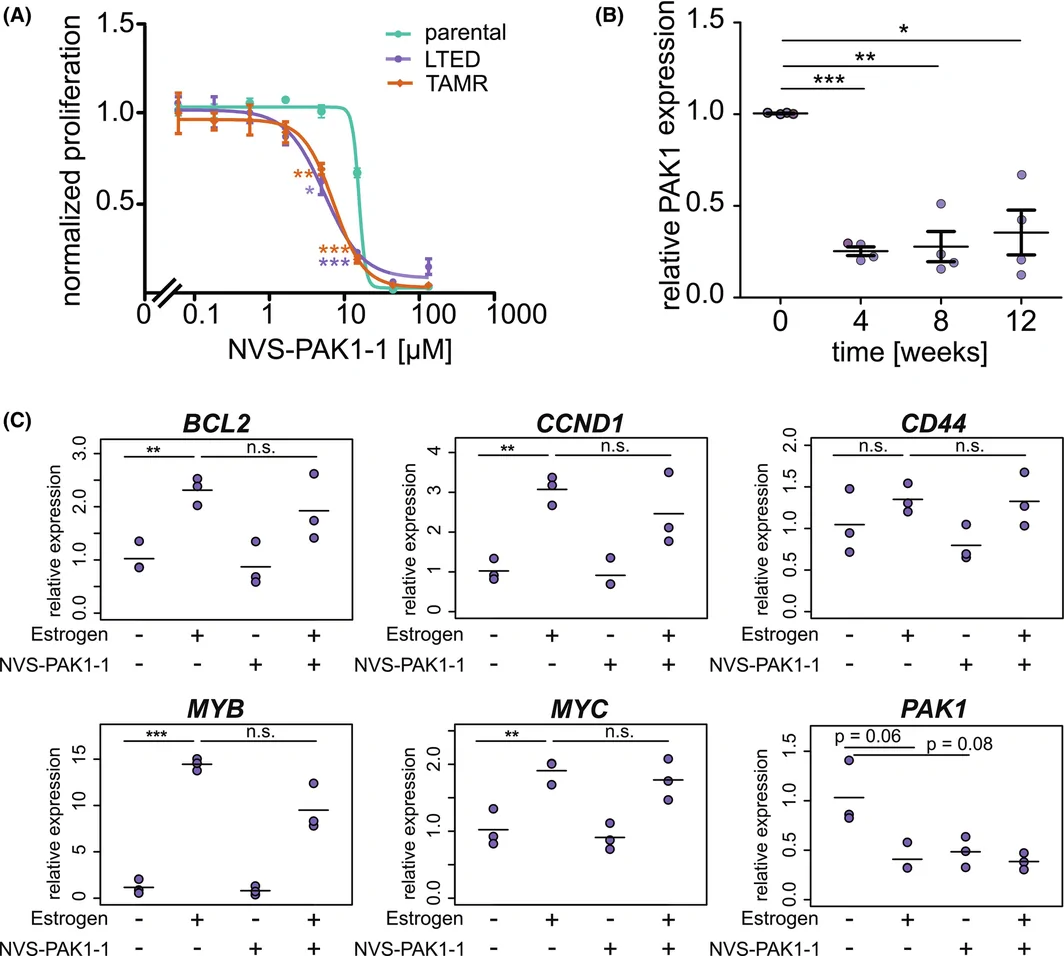
PAK1 inhibitor treatment and *PAK1* expression upon re‐exposure to estrogen in MCF7 parental, long‐term estrogen deprived (LTED) and tamoxifen‐resistant (TAMR) cells. (A) MCF7 parental, LTED and TAMR cells were treated with different concentrations of PAK1 inhibitor NVS‐PAK1‐1 or DMSO control. Proliferation was determined by counting the numbers of nuclei in alive cells at day 7 and related to respective numbers at day 1, relative to the DMSO control (*n* = 3), 2‐sided *t*‐test, * = *P* < 0.05, ** = *P* < 0.01, *** = *P* < 0.001. Error bars indicate standard deviation (SD). (B) Estrogen was re‐introduced into LTED medium for 4, 8, or 12 weeks and *PAK1* expression in MCF7 LTED cells was measured via RT‐qPCR. *PAK1* expression is shown relative to medium without estrogen (*n* = 4), 2‐sided *t*‐test, * = *P* < 0.05, ** = *P* < 0.01, *** = *P* < 0.001. Error bars indicate SD. (C) MCF7 LTED cells were treated with 6 μM NVS‐PAK1‐1 or DMSO for 60 min. Subsequently, the culture medium was exchanged for either LTED medium (without estrogen) or full growth medium (with estrogen), and culture continued for another 24 h. Then, expression of the indicated genes was measured by RT‐qPCR. Expression was normalized to *ACTB* and *PUM1* housekeeping genes and the DMSO control sample without estrogen. Statistical testing was performed using 2‐sided *t*‐test, * = *P* < 0.05, ** = *P* < 0.01, *** = *P* < 0.001, ns = not significant (n = 3).

To test whether PAK1 influences the activity of the estrogen receptor (ERα) and triggers estrogen‐like signaling, we treated MCF7 LTED cells again with the PAK1 inhibitor NVS‐PAK1‐1. Subsequently, we stimulated the cells with estrogen for 24 h and analyzed the expression of selected estrogen‐responsive genes. Most of these genes were expressed at significantly higher levels one‐day poststimulation, confirming their regulation by estrogen (Fig. 2C). In contrast, PAK1 inhibition did not affect ERα target gene expression irrespective of estrogen supply, suggesting that PAK1 does not trigger ERα signaling in this setting. In line with the results from our long‐term experiment (Fig. 2B), we observed reduced *PAK1* expression already upon short‐time exposure to estrogen (Fig. 2C). Unexpectedly, blocking PAK1 activity with the small molecule inhibitor reduced *PAK1* expression even in the absence of estrogen, suggesting that active PAK1 might regulate its own gene expression.

Here, we have shown that both endocrine therapy‐resistant cell models were more sensitive to PAK1‐inhibition compared to parental cells, suggesting that PAK1 is required for proliferation and survival when estrogen signaling is abrogated by either depletion of estrogen (i.e., aromatase inhibition) or by selective modulation of estrogen receptor signaling (i.e., tamoxifen treatment).

### EGF stimulation triggers prolonged activation of downstream pathways and further enhances PAK1 activity in LTED and TAMR cell line models

In a previous study, we had shown that MCF7 expresses moderate levels of EGFR and responds to EGF stimulation with a fast and transient activation mostly of RAS–RAF–ERK signaling [54]. Furthermore, an activating mutation in *PIK3CA* (i.e., E545K) that is present in MCF7 triggers a prominent basal activation also of the PI3K‐AKT–mTOR pathway [54]. In addition to MAPK and AKT signaling, EGFR has been described to activate PAK1 via RAC1 or NCK [55, 56]. As regulation of PAK1 via RAC1 has been associated with tamoxifen resistance [52], we hypothesized that EGF stimulation might modulate PAK1 activity also in our endocrine resistance models. To test this, we performed a time‐resolved experiment with EGF stimulation to assess PAK1 signaling and pathway activation over time, again using an MS‐based total proteomic and phosphoproteomic readout (Data S7 and S8).

The activation of pathways downstream of EGFR is commonly estimated based on the phosphorylation levels of classical regulatory sites in selected proteins involved in EGFR signaling, such as *MAPK3*/ERK1 T202/Y204 and *MAPK1*/ERK2 T185/Y187 [57]. Indeed, the phosphorylation levels at these sites were strongly upregulated upon EGF stimulation as compared to growth factor‐deprived cells (Fig. S2). We then utilized the phosphoproteome data to estimate the dynamic changes in kinase activities within every cell model and at each time point compared to the unstimulated control condition (Data S9). In agreement with the dynamics of selected classical regulatory sites (Fig. S2), we observed increased activities particularly of kinases in the MAPK/AKT/mTOR/S6K signaling pathways downstream of EGFR in response to EGF stimulation in parental, LTED, as well as TAMR cells (Fig. 3A).

**Fig. 3:**
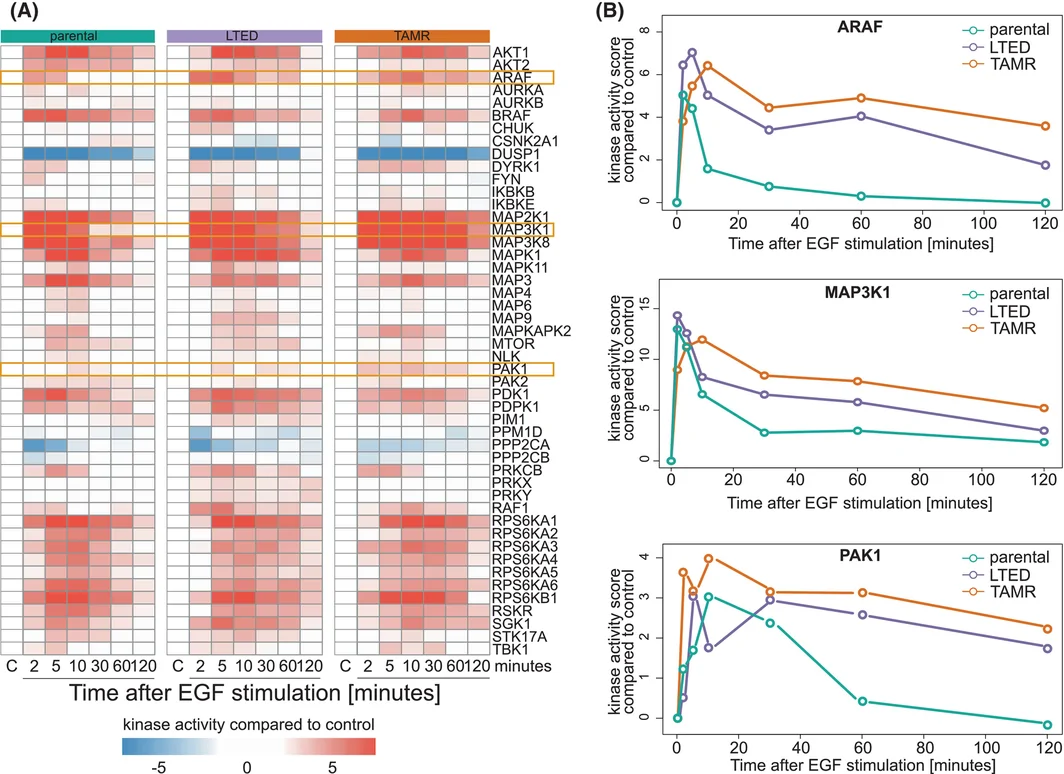
Phosphoproteomic changes in MCF7 parental, long‐term estrogen deprived (LTED) and tamoxifen‐resistant (TAMR) cell lines follow different kinetics upon EGF stimulation. MCF7 parental, LTED and TAMR cells were serum‐starved for 24 h to deplete growth factors, followed by stimulation with 5 nm EGF for indicated time points or left non‐stimulated (control = C). Cells were lysed at the indicated time points after addition of EGF. Protein clean‐up and digestion was performed using SP3 [30] with 500 μg protein input. Peptides were enriched for phosphorylation, and both the total proteome and phosphoproteome fractions were analyzed by DIA LC–MS/MS. Proteins were identified using Spectronaut (v17), and the localization cut‐off was set to 0.95 for phosphosite identification with the PeptideCollapse plugin in Perseus. Statistical analysis of phosphosite intensities compared to timepoint 0 min was performed using the eBayes function of limma (*n* = 3). Kinase activities were calculated on the t‐statistics of the respective comparisons, using decoupleR and Omnipath [40]. (A) Kinase activities within each condition compared to the respective unstimulated condition (timepoint 0). (B) Activities of PAK1, RAF1, and MAP3K1 kinases over time.

Structural changes in the EGFR that are induced upon ligand‐binding have been associated with differential duration of growth factor activity and different phenotypic effects these induce [58]. The duration of signaling pathways downstream of EGFR, like MAPK, is further regulated by several other mechanisms, including positive and negative feedback loops, the availability of scaffold proteins, and cross‐talk between different MAPK pathways [59, 60]. Even minor changes in the duration of activity of these proteins can have substantial biological effects on cellular proliferation and survival dynamics [59]. Therefore, we next assessed the dynamics of downstream EGF responses in the two endocrine resistance models as compared to the parental cells. To this end, we applied a time‐resolved analysis to reveal differences in the activation dynamics of phosphosites and their respective kinases relative to the parental cell line. There, two‐way ANOVA identified 279 phosphosites with significantly different dynamics upon EGF stimulation (Fig. S3A). Most of the sites showed prolonged phosphorylation in LTED and TAMR with increased intensities lasting for 60 or even 120‐min post‐EGF stimulation, while most of these phosphosites returned back to baseline intensities within 30 min in the parental cell line (Fig. S3A). Reactome pathway analysis revealed that these differentially phosphorylated sites prominently mapped to proteins related to receptor tyrosine kinase and MAPK signaling (Fig. S3B), indicating potentially prolonged signaling of receptor tyrosine kinase and MAPK pathways. Sixty of these phosphosites had an upstream targeting kinase annotated in the Omnipath database. Specifically, ARAF, BRAF, MAP2K1, MAPK3, MAPK1, PDK1, AKT1, RPS6KB1, PRS6KA3, and RPS6KA1 were overrepresented as targeting kinases for this set of phosphosites with significantly altered time‐resolved dynamics in the resistance models, indicating mostly prolonged activation dynamics (Fig. S4).

Furthermore, PAK1 remained at higher activation levels in both LTED and TAMR even 120 min after EGF stimulation, while its activity in parental cells was back to the basal level after 60 min. Similarly, the activation of ARAF and MAP3K1 was extended in the LTED and TAMR cells (Fig. 3B), further supporting a role of MAPK signaling in resistance. The consistent finding of prolonged protein phosphorylation as well as kinase activation suggests that likely reduced activities of negative feedback loops ensure longer lasting signal activities in the endocrine therapy‐resistant cell lines, potentially contributing to altered phenotypes, including endocrine resistance.

### LTED and TAMR show similar kinase activity adaptations and increased PAK1 activity

Thus far, we have investigated time‐resolved kinase activity dynamics relative to the unstimulated control condition within each cell model. Using the same datasets, we next compared the kinase activities of LTED and TAMR cells relative to the parental cells for every matching time point of EGF stimulation (Fig. 4A; Data S10). The sets of consistently deregulated kinases, with an absolute activity score > 2 compared to parental, largely overlapped in LTED and TAMR across all time points of EGF stimulation. These kinases included more active PAK1, BRAF, as well as several members of the RPS6K family (Fig. 4A). The phosphatase DUSP1 was less active in both resistance models compared to parental, especially at the later time points (Fig. 4A), which might explain the prolonged signaling duration we observed [61]. The degree of kinase activity deregulation was highly similar between LTED and TAMR with a Pearson correlation coefficient of 0.701 (Fig. 4B). In contrast, individual kinase activities and their respective protein abundance were largely not correlated (Fig. S5), highlighting the importance of assessing protein or pathway activities rather than gene or protein expression only.

**Fig. 4:**
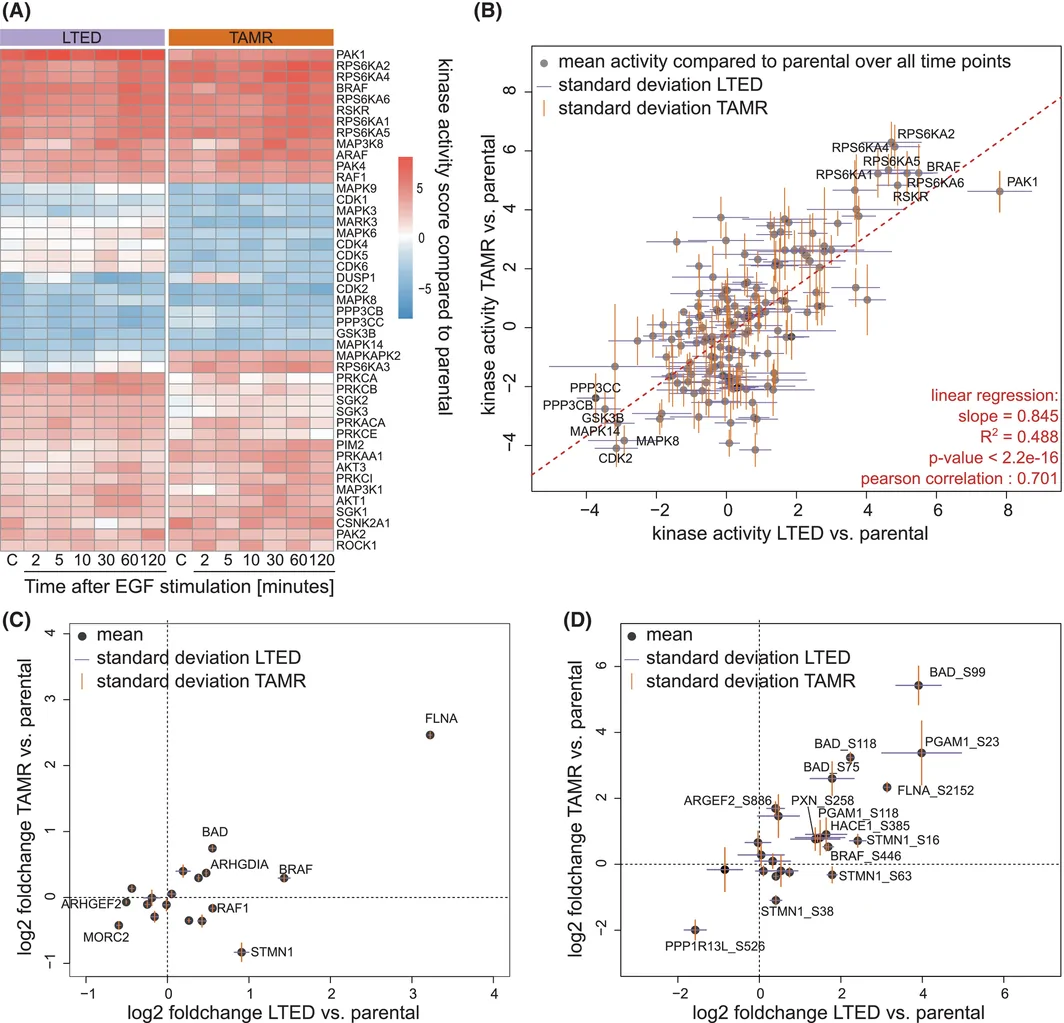
Kinase activities in MCF7 long‐term estrogen deprived (LTED) and tamoxifen‐resistant (TAMR) are distinct from the parental cells and consistent over the time course of EGF stimulation. MCF7 parental, LTED, and TAMR cells were serum‐starved for 24 h, followed by stimulation with 5 nm EGF or cultivated without stimulation (control = C) (*n* = 3). Cells were lysed at the indicated time points after stimulation. Protein clean‐up and digestion was performed using SP3 [30] with 500 μg protein input. Peptides were enriched for phosphorylation, and both the total proteome and phosphoproteome fractions were analyzed by DIA LC–MS/MS. Proteins were identified using Spectronaut (v17), and the localization cut‐off was set to 0.95 for phosphosite identification with the PeptideCollapse plugin in Perseus. Statistical analysis of the phosphosite intensities was performed using the eBayes function of limma. Kinase activities were calculated on the t‐statistics of each time point compared to the matched time point in the parental cells, using decoupleR and Omnipath. (A) Kinases with absolute activity scores > 2 in long‐term estrogen‐deprived (LTED) and tamoxifen‐resistant (TAMR) compared to parental cells at each time point of EGF stimulation. (B) Mean and standard deviation of all kinases compared to parental cells over all time points. (C) Log2 fold changes of total proteome protein abundance and phosphosite intensities of known PAK1 substrates in LTED compared to parental. Shown are mean ± SEM over all time points. Proteins with an absolute log2 fold change > 0.5 and a significant difference to parental MCF7 are indicated. (D) Log2 fold changes of protein abundance and phosphosite intensities of known PAK1 substrates in LTED vs. parental and TAMR compared to parental. Shown are mean ± SD of all time points. Phosphosites with an absolute log2 fold change > 1 and a significant difference to parental cells are labeled.

Again, PAK1 was found to be the most activated kinase in LTED compared to parental cells (Fig. 4B). Interestingly, while PAK1 was only slightly more active in TAMR under normal growth conditions (score = 0.52, see Fig. 1D), it was four times more active in the serum‐deprived setting (Fig. 4B). PAK1 is known to be activated by EGFR signaling especially under nutrient stress [55]. While TAMR cells clearly depend on PAK1 for proliferation also in complete growth medium (Fig. 2A), serum starvation thus might be required for full activation of PAK1.

To zoom in on potential mechanisms downstream of PAK1 that might explain estrogen signaling‐independent survival and proliferation, we next focused on the phosphorylation levels of known PAK1‐substrates, as annotated in the Omnipath database (Data S11) [40]. Highly elevated protein levels of Filamin A (FLNA) were detected in both LTED and TAMR cells (Fig. 4C), and the protein was also more strongly phosphorylated, specifically at serine residue S2152 (Fig. 4D). Phosphorylation at this site by PAK1 has been described as part of a positive feedback loop where the interaction between FLNA and PAK1 enhances PAK1 kinase activity [62]. Also, in line with our findings, FLNA has been associated with various cellular processes, including cell adhesion, proliferation, and DNA repair [62] and has previously been implicated as a driver in chemotherapy resistance by activating MEK–ERK signaling [63]. The pro‐apoptotic protein BCL2 Associated Agonist of Cell Death (BAD) was significantly more phosphorylated at residues S99, S118, and S75 in endocrine‐resistant cells. Especially in the TAMR condition, a more than 30‐fold higher intensity of S99‐phosphorylated BAD was measured compared to parental cells (Fig. 4D). All three phosphorylation sites have been reported to inhibit the pro‐apoptotic function of BAD [64], suggesting a potential involvement of BAD also in estrogen‐independent survival of endocrine therapy‐resistant cells.

In summary, the kinase activity landscapes we uncovered in response to EGF stimulation were mostly similar in LTED and TAMR models. PAK1 was consistently among the most strongly activated kinases in both resistance models; its activity was further increased upon EGF stimulation and demonstrated prolonged activation compared to parental cells. The dependence of endocrine therapy‐resistant cells on PAK1 signaling for survival and proliferation might also be related to increased phosphorylation of downstream substrates, like FLNA and BAD.

### Inhibition of PAK1 indicates distinct wiring of downstream pathways in the two resistance models

To further elucidate the signaling network downstream of PAK1 in the LTED and TAMR resistance models and to uncover how it might trigger estrogen‐independent survival and proliferation, we acquired phosphoproteome data after short‐term PAK1 inhibition. LTED and TAMR cells were treated with the PAK1‐specific inhibitor NVS‐PAK1‐1, at a concentration close to the previously determined IC50 for LTED and TAMR cells (see Fig. 2). As expected, no significant changes were detectable in the total proteomes after one hour of PAK1 inhibition, as compared to the DMSO control (Fig. S6A; Data S13 and S14). In contrast, strong effects were visible in the phosphoproteomic data (Data S15, S16 and S17). MAPK1, MAPK3, MAPK8, and AKT showed consistently increased activities upon PAK1 inhibition in both cell models (Fig. 5A), supporting an involvement of PAK1 in a negative feedback loop between EGF and the MAPK/AKT pathways [65]. In contrast, decreased activities of the kinases PAK1, CDK17, and most prominently, PAK2 were observed in both LTED and TAMR upon short‐term PAK1 inhibition (Fig. 5A). While the strong repression of PAK2 could potentially hint at an off‐target effect of the PAK1 inhibitor, several points speak against an involvement also of PAK2. Firstly, inspection of residues that are specifically phosphorylated by PAK2 revealed that its predicted lower activity was mostly associated with decreased phosphorylation at the activating auto‐phosphorylation site S141 [66] of PAK2 [66] (Fig. S6B). This might support a direct interaction between PAK1 and PAK2, which has indeed been described before [67], rather than an off‐target effect of the inhibitor. Moreover, serine 518 (S518) in NF2 is annotated as a PAK2‐specific phosphorylation site in Omnipath and was repressed in both cell models (Fig. S6B); however, it has been reported to be phosphorylated also by PAK1 [68] and by PRKACA [69]. As PRKACA and PAK1 activities were strongly reduced in the LTED cells (compare Fig. 5A), diminished phosphorylation of NF2 might thus be triggered by lowered activities of PAK1 and/or PRKACA there. Additionally, the NVS‐PAK1‐1 inhibitor has been reported as a selective PAK1 inhibitor having an almost 60‐fold higher selectivity for PAK1 over PAK2 [70]. And finally, activation of PAK1, however, not of PAK2 has previously been associated with breast cancer, and there prominently with the luminal subtypes [71]. Together, those findings support the concept that PAK1, and not its paralogue PAK2, could be a driver of endocrine resistance in our cell line models.

**Fig. 5:**
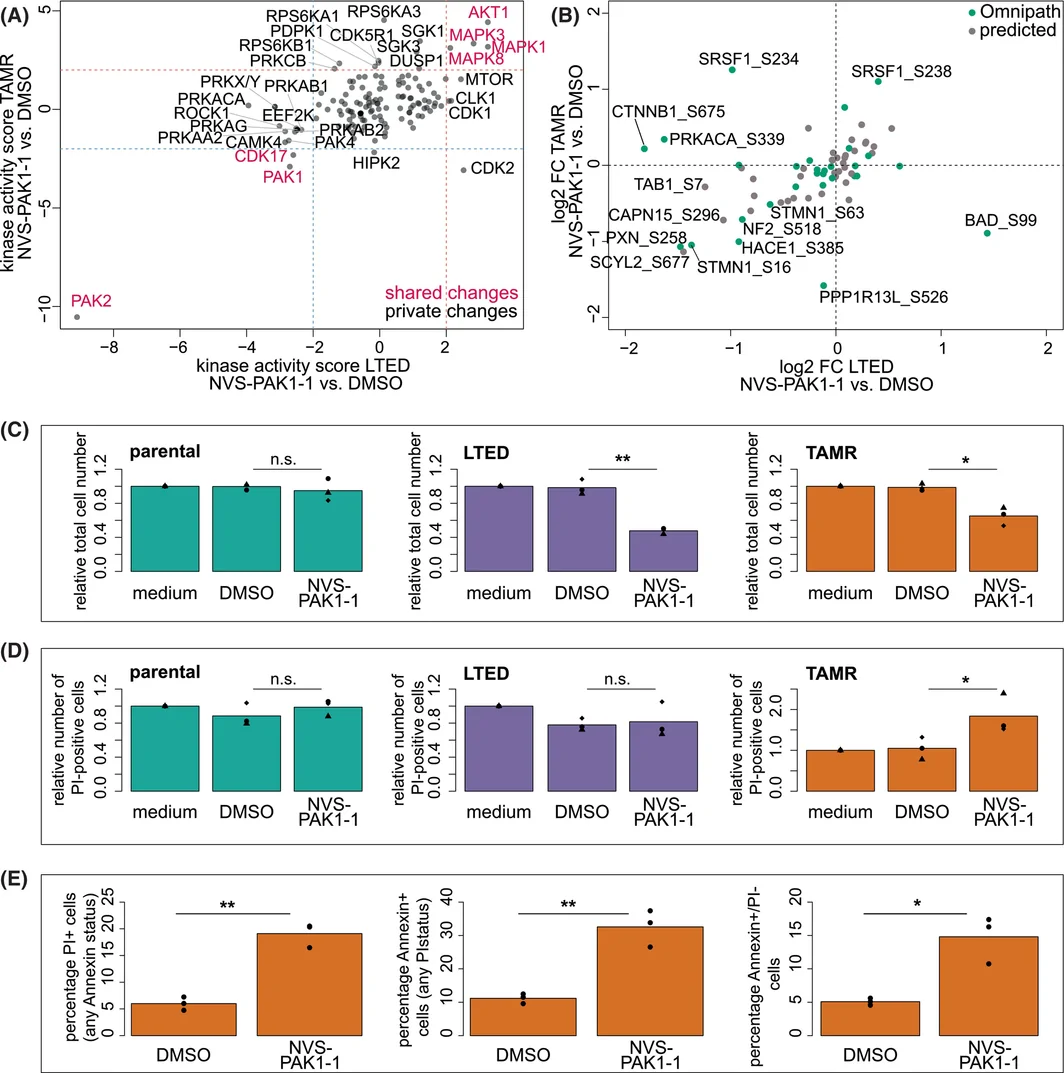
Immediate downstream effects of PAK1 inhibition in MCF7 long‐term estrogen deprived (LTED) and tamoxifen‐resistant (TAMR) cells. MCF7 parental, LTED, and TAMR cells were treated with 6 μM PAK1 inhibitor NVS‐PAK1‐1 for 1 h. Proteins were prepared for LC–MS/MS analysis by automated SP3 [31] and phospho‐enrichment. Total proteome and phosphoproteome fractions were analyzed by LC–MS/MS for 120 min in DIA mode. Peptides and proteins were identified and quantified using Spectronaut (v17). Phosphorylated peptides were collapsed to the site‐level using the Perseus plug‐in PeptideCollapse with a localization cut‐off at 0.95. Statistical comparison was performed using the eBayes function of limma (v. 3.58.1). Kinase activities were calculated on the t‐statistics of the respective comparison, using decoupleR and Omnipath. (A) Kinase activity scores upon PAK1 inhibition for 1 h in LTED and TAMR compared to the DMSO control. (B) Known PAK1 substrates were retrieved from the Omnipath database [40], predicted PAK1 substrates were obtained from Johnson et al. [74] by filtering for phosphosites with PAK1 in at least the 98th percentile and ranking higher than 5. Shown are log2 fold changes of known and predicted PAK1 substrates' intensities in the PAK1‐inhibited condition compared to the DMSO control in LTED and TAMR, respectively. (C,D) MCF7 parental, LTED, and TAMR cells were treated with 6 μM NVS‐PAK1‐1 for 7 days. Proliferation was measured by Hoechst staining. Dead cells were stained with Propidium iodide (PI). The number of cells was compared to day 1 and further normalized by the normal growth medium. Statistics were performed using a one‐sided unpaired t‐test to test for fewer living cells or more dead cells, respectively. (E) MCF7 TAMR cells were treated with 6 μM NVS‐PAK1‐1 for 7 days. Then, cells were stained with Hoechst, PI, and Annexin V‐FITC. The proportion of dead cells was assessed based on the number of PI‐positive cells (left panel). The proportion of any apoptotic cells was assessed based on the number of Annexin V FITC‐positive cells irrespective of PI staining (middle panel). The proportion of early apoptotic cells was assessed based on the number of Annexin V FITC‐positive and PI‐negative cells (right panel). Statistics (in C–E) were performed using one‐sided unpaired t‐tests, * = *P* < 0.05, ** = *P* < 0.01, *** = *P* < 0.001, ns = not significant (*n* = 3).

The effect of PAK1‐inhibition further revealed that phosphorylation of several PAK1 substrates annotated in the Omnipath database, specifically PXN (S258) and STMN1 (S16, S63), was strongly decreased in both LTED and TAMR (Fig. 5B), suggesting their direct regulation by PAK1 in both resistance models. Databases of known kinase‐substrate networks, such as Omnipath [40], rely on experimentally validated relationships and might therefore miss out on so far unknown interactions. Only about 5% of identified phosphosites have known upstream kinases and well‐characterized functions [72]. As a result, current phosphoproteome analyses are restricted to a few kinases and their annotated substrates. New tools for the prediction of kinase/substrate and phosphosite/functionality interfaces promise to broaden the application spectrum of phosphoproteomics [72, 73, 74, 75, 76]. To identify new potential downstream substrates of PAK1, we made use of the target site prediction tool published by Johnson et al. [74] (Data S12). Several of the PAK1 substrates predicted there, such as Table 1 (S7), CAPN15 (S296), and SCYL2 (S677), indeed showed decreased phosphorylation in response to PAK1 inhibition (Fig. 5B), which supports them as putative PAK1 substrates, at least in the context of our LTED and TAMR models.

Further, PAK1 inhibition exerted differential effects in LTED vs. TAMR, such as a decrease in phosphorylation of PRKACA (S339) and CTNNB1 (S675) in LTED only (Fig. 5B), suggesting that PAK1 potentially acts via activating these known pro‐proliferative proteins specifically in the LTED context. In contrast, inhibition of PAK1 decreased phosphorylation of BAD at S99 exclusively in the TAMR cells (Fig. 5B), which is in line with a previous study which had associated this phosphosite with tamoxifen resistance [77]. Hence, our data suggest that in TAMR cells PAK1 might act through inhibition of BAD to promote estrogen‐independent survival.

To test the phenotypes associated with proliferation and survival, we treated MCF7 parental, LTED and TAMR cells with the PAK1‐inhibitor and measured the total number of cells, as well as the number of dead cells after 7 days. Consistent with the data shown in Fig. 2A, the proliferation of parental MCF7 cells remained unaffected by PAK1 inhibition while, as expected, proliferation was reduced by 40–50% in both LTED and TAMR cells (Fig. 5C). However, the number of dead, PI‐positive, cells was significantly increased by PAK1‐inhibition only in the TAMR condition whereas cell death seemed to be unaffected in LTED cells as well as in the DMSO control (Fig. 5D). Our phosphoproteomic data had shown reduced phosphorylation of the pro‐apoptotic BAD protein at the inhibitory position S99 upon PAK1 inhibition specifically in the MCF7 TAMR cells. We thus hypothesized that the observed increase in the percentage of dead cells in this condition might correspond to increased apoptosis. To test this, we inhibited PAK1 in TAMR cells and stained with both Annexin V‐FITC and PI, to specifically characterize apoptotic cell death. We again observed that after 7 days of PAK1 inhibition a significantly higher proportion of TAMR cells was PI‐positive, that is, nonviable (Fig. 5E, left panel). More than 30% of TAMR cells were positive for Annexin V, indicating that these cells were undergoing apoptosis (middle panel), with around half of them being early apoptotic (Annexin V+/PI‐, right panel). Hence, the proportion of apoptotic cells was significantly increased upon PAK1 inhibition in TAMR cells. This finding strongly suggests that PAK1 protects TAMR cells from apoptosis, presumably through phosphorylation of BAD.

## Discussion

As PAK1 is a known driver in acquired tamoxifen resistance [15, 16, 52] and since we saw high levels and strong activation of PAK1 particularly in our MCF7 LTED model, we hypothesized that PAK1 might be involved also in resistance to estrogen deprivation by aromatase inhibitors. While PAK1 activation indeed contributed to endocrine therapy resistance also in the LTED cell line model, our data suggest that, although both LTED and tamoxifen‐resistant models rely on PAK1, the functional outcomes are mediated by different substrate networks. These findings likely reflect unique adaptive responses to estrogen deprivation versus tamoxifen antagonism (Fig. S7): While PAK1 activation in LTED prominently drove proliferative signaling through phosphorylation of CTNNB1, its role in TAMR was more anti‐apoptotic, marked by strong phosphorylation of BAD. This implies that PAK1 supports endocrine resistance via distinct mechanisms, that is, mitogenic in LTED and survival‐promoting in TAMR, highlighting the need for context‐specific therapeutic strategies within ER+ breast cancer subtypes. Our time‐resolved measurements further suggested that PAK1‐activities were mediated, at least in part, by prolonged activation of downstream signaling networks, prominently of MAPK signaling.

Resistance mechanisms in Erα‐positive breast cancer have been studied for decades [78] and multiple studies have elucidated potential resistance mechanisms, including estrogen‐independent activation of ERα and the activation of alternative survival pathways via, for example, EGFR, HER2, MAPK, and PI3K/AKT signaling [14, 16, 79, 80, 81, 82, 83] and metabolic rewiring [84]. Collectively, resistance mechanisms appear to be manifold and might also derive from stochastic clonality during the outgrowth of resistant cells [85, 86]. Generally, resistance acquisition is thus a highly individual and heterogeneous process. While we identified the reliance on PAK1‐activity in two models of endocrine resistance, this should not be considered a universal resistance mechanism.

Hence, our findings on PAK1 as a novel potential player add to the network of endocrine resistance mechanisms. In the era of precision oncology, the identification of individual resistance mechanisms to specific treatments is key toward the molecularly informed guidance of second‐line treatment decisions. Along this line, inhibition of PAK1 could potentially serve as one future strategy to revert or even prevent endocrine therapy resistance in breast cancer. However, further *in vitro* and *in vivo* studies are required to validate PAK1 as a viable therapeutic target.

## Conclusion

In summary, we identified PAK1 as a regulator of divergent downstream signaling networks in two models of endocrine‐resistant luminal breast cancer, thereby suggesting PAK1 inhibition as a potential clinically relevant therapeutic strategy.

## Conflicts of interests

The authors declare no conflicts of interest.

## Author contributions

LS, JM, LB, DH, CK, and SW conceived the study. LS, JM, LB, LF, AW, SK, and SB designed and/or performed experiments. LS, and EV analyzed and visualized the data. LS, CK, and SW wrote the manuscript. All authors read and approved the final manuscript.

## Supporting information


**Fig. S1.** Total proteome adaptations in MCF7 long‐term estrogen deprived (LTED) and tamoxifen resistant (TAMR) cells.
**Fig. S2.** Phosphorylation dynamics of phosphosites known to be regulated by EGF signaling quantified in MCF7 parental, long‐term estrogen deprived (LTED), and tamoxifen resistant (TAMR) cells.
**Fig. S3.** Two‐way ANOVA analysis of phosphosites over time during EGF stimulation of MCF7 parental, long‐term estrogen deprived (LTED), and tamoxifen resistant (TAMR) cells.
**Fig. S4.** Kinase annotation of significant phosphosites in MCF7 parental, long‐term estrogen deprived (LTED) and tamoxifen resistant (TAMR) cells from two‐way ANOVA over time.
**Fig. S5.** Comparison of kinase activities and expression levels in long‐term estrogen deprived (LTED) and tamoxifen resistant (TAMR) MCF7 cells, in comparison to the parental cells.
**Fig. S6.** Proteome analysis following PAK1‐inhibitor treatment of MCF7 parental, long‐term estrogen deprived (LTED), and tamoxifen resistant (TAMR) cells.
**Fig. S7.** PAK1 triggers resistance in MCF7 tamoxifen resistant (TAMR) and long‐term estrogen deprived (LTED) resistance models, however, via different downstream mechanisms.

**Data S1.** Total proteome data of MCF7 parental, long‐term estrogen deprived (LTED) and tamoxifen resistant (TAMR) LTED cells in standard growth condition with complete medium.

**Data S2.** Phosphoproteome data of MCF7 parental, long‐term estrogen deprived (LTED), and tamoxifen resistant (TAMR) cells in standard growth condition with complete medium.

**Data S3.** Total proteome statistics of MCF7 long‐term estrogen deprived (LTED) and tamoxifen resistant (TAMR) vs. parental cells, respectively.

**Data S4.** Reactome pathway overrepresentation analysis of total proteomes measured in MCF7 parental, long‐term estrogen deprived (LTED) and tamoxifen resistant (TAMR) cells.

**Data S5.** Phosphoproteome statistics of MCF7 long‐term estrogen deprived (LTED) and tamoxifen resistant (TAMR) vs. parental cells, respectively.

**Data S6.** Kinase activities of MCF7 long‐term estrogen deprived (LTED) and tamoxifen resistant (TAMR) vs. parental cells, respectively.

**Data S7.** Total proteome result of EGF‐stimulated MCF7 parental, long‐term estrogen deprived (LTED) and tamoxifen resistant (TAMR) cells.

**Data S8.** Phosphoproteome result of EGF‐stimulated MCF7 parental, long‐term estrogen deprived (LTED) and tamoxifen resistant (TAMR) cells.

**Data S9.** Kinase activities of EGF stimulated MCF7 parental, long‐term estrogen deprived (LTED) and tamoxifen resistant (TAMR) cells compared to unstimulated cells.

**Data S10.** Kinase activities of EGF‐stimulated MCF7 long‐term estrogen deprived (LTED) and tamoxifen resistant (TAMR) vs. parental cells at each time point.

**Data S11.** Annotated PAK1 target sites in the Omnipath database.

**Data S12.** Predicted PAK1 target sites.

**Data S13.** Total proteome result of PAK1‐inhibited MCF7 parental, long‐term estrogen deprived (LTED) and tamoxifen resistant (TAMR) cells.

**Data S14.** Total proteome statistics of PAK1‐inhibited MCF7 parental, long‐term estrogen deprived (LTED), and tamoxifen resistant (TAMR) vs. respective DMSO‐treated control cells.

**Data S15.** Phosphoproteome results of PAK1‐inhibitor‐ and DMSO‐treated MCF7 parental, long‐term estrogen deprived (LTED) and tamoxifen resistant (TAMR) cells.

**Data S16.** Phosphoproteome statistics of PAK1‐inhibited MCF7 parental, long‐term estrogen deprived (LTED), and tamoxifen resistant (TAMR) vs. respective DMSO‐treated control cells.

**Data S17.** Kinase activities of PAK1‐inhibited MCF7 parental, long‐term estrogen deprived (LTED), and tamoxifen resistant (TAMR) vs. respective DMSO‐treated control cells.

## Data Availability

The mass spectrometry proteomics data have been deposited to the ProteomeXchange Consortium via the PRIDE [87] partner repository with the dataset identifier PXD063393. All data files (normalized protein or phosphosite data sets, statistics outputs), as well as the code used for data quality control, normalization and creating the plots, are available on GitHub (https://github.com/schwarlu1691995/PAK1_manuscript).
