# Supplementary material for: PAK1 activation drives divergent resistance mechanisms to aromatase inhibition and tamoxifen in a luminal: A breast cancer model
Source: Mol Oncol. 2026 Jun 5;20(11):2764–83. doi: 10.1002/1878-0261.70286 (PMC13399542; doi:10.1002/1878-0261.70286)
Supplement: Supplementary file 1 [file MOL2-20-2764-s008.docx]

## Supplementary Figures and Figure legends

**Suppl. Figure 1: Total proteome adaptations in MCF7 long-term estrogen deprived (LTED) and tamoxifen resistant (TAMR) cells.** MCF7 parental, LTED, and TAMR cells were lysed in RIPA buffer, followed by 30 minutes of DNase/Benzonase treatment on ice. Protein clean-up and digestion was performed using manual SP3 with 500 µg protein input. Peptides were enriched for phosphorylation and both the total and phosphoproteome fractions were analyzed by LC-MS/MS for 120 minutes in DIA mode. Peptides and proteins were identified and quantified using Spectronaut (v17) in directDIA+ mode. (A) Numbers of identified protein groups in three biological replicates. The red dashed line indicates the number of proteins that were identified in all replicates of all conditions. (B) Overlap of protein groups identified in all biological replicates (n=3). (C, D) Statistical analysis was performed using the eBayes function of the limma R package. Reactome pathway overrepresentation analysis was performed, using a threshold of fold-change > 2 (UP) or < 0.5 (DOWN) and an adjusted p-value < 0.05. Log2 fold-changes (log2 FC) and -log10 adjusted p-values are shown for all proteins in LTED and TAMR compared to parental cells. Proteins involved in the significantly overrepresented pathways “cholesterol biosynthesis” and “gluconeogenesis” are highlighted in LTED (C) and TAMR (D), respectively.

**Suppl. Figure 2: Phosphorylation dynamics of phosphosites known to be regulated by EGF signaling quantified in MCF7 parental, long-term estrogen deprived (LTED), and tamoxifen resistant (TAMR) cells.** Time course of selected phosphosites involved in EGF signaling (according to PTMSigDB^72^) in response to EGF stimulation for indicated time points, compared to the non-stimulated control (C). Effects of phosphorylation events on protein activities (inhibiting or activating) are indicated. The color represents the mean log2 fold-changes (log2 FC) relative to the control without stimulation (n=3).

**Suppl. Figure 3: Two-way ANOVA analysis of phosphosites over time during EGF stimulation of MCF7 parental, long-term estrogen deprived (LTED), and tamoxifen resistant (TAMR) cells.** (A) Z-scaled intensities of phosphosites found the be significantly different over time and between parental, LTED, and TAMR cells after stimulation with EGF for the indicated time points, compared to the respective non-stimulated control (C) in a 2-way ANOVA analysis. (B) Reactome gene set enrichment of all proteins found phosphorylated in A. Adjusted p-values (p.adjust) and numbers of deregulated proteins (counts) within a respective Reactome gene set are indicated.

**Suppl. Figure 4: Kinase annotation of significant phosphosites in MCF7 parental, long-term estrogen deprived (LTED) and tamoxifen resistant (TAMR) cells from two-way ANOVA over time.** Overrepresentation analysis of phosphosites from Suppl. Figure 3 with a targeting kinase annotated in Omnipath kinases assigned to the respective sites. Shown are the proteins and phosphosites with Z-scored phophosite intensities at the indicated time points after EGF-stimulation, and the annotated targeting kinases. This revealed significant overrepresentation of the ten indicated kinases, after stimulation of cells with EGF for indicated time points, compared to the non-stimulated control (C).

**Suppl. Figure 5: Comparison of kinase activities and expression levels in long-term estrogen deprived (LTED) and tamoxifen resistant (TAMR) MCF7 cells, in comparison to the parental cells.** Mean kinase activity in LTED (A) and TAMR (R) compared to parental MCF7 over all time-points and mean kinase abundance levels as log2 fold-change (log2 FC) compared to parental cells over all time point (n=3).

**Suppl. Figure 6: Proteome analysis following PAK1-inhibitor treatment of MCF7 parental, long-term estrogen deprived (LTED), and tamoxifen resistant (TAMR) cells.** MCF7 parental, LTED, and TAMR cells were treated with PAK1 inhibitor NVS-PAK1-1 for 60 minutes, followed by LC-MS/MS analysis (n=3). (A) Z-scored heatmap and unsupervised hierarchical clustering of protein quantifications. (B) t-statistics of annotated PAK2 target sites {Turei, 2021 #20532} in PAK1 inhibitor NVS-PAK1-1 vs. DMSO treated LTED and TAMR cells.

**Suppl Figure 7: PAK1 triggers resistance in MCF7 long-term estrogen deprived (LTED) and tamoxifen resistance (TAMR) models, however, via different downstream mechanisms.** Endocrine therapies for ER+ breast cancer often face resistance. Using cellular models for resistance to aromatase inhibition (LTED) and tamoxifen (TAMR), quantitative phosphoproteomic mass spectrometry, and computational and experimental analysis, we found that PAK1 is a key mediator of resistance in both LTED and TAMR resistance models, however, triggered cell proliferation and blocked apoptosis in these models, respectively. E2 17-β-estradiol, ERα estrogen-receptor alpha, ERE estrogen responsive element, Tam 4-hydroxytamoxifen.

## Legends for Supplementary Data:

**Suppl. Data 1: Total proteome data of MCF7 parental, long-term estrogen deprived (LTED) and tamoxifen resistant (TAMR) LTED cells in standard growth condition with complete medium.** MCF7 parental, LTED, and TAMR cells were cultivated in complete medium, lysed in RIPA buffer, followed by 30 minutes of DNase/Benzonase treatment on ice. Protein clean-up and digestion was performed using manual SP3 with 500 µg protein input. Peptides were enriched for phosphorylation and both the full and phosphoproteome fractions were analyzed by LC-MS/MS for 120 minutes in DIA mode. Peptides and proteins were identified and quantified using Spectronaut (v17) in directDIA+ mode. (n=3)

**Suppl. Data 2: Phosphoproteome data of MCF7 parental, long-term estrogen deprived (LTED), and tamoxifen resistant (TAMR) cells in standard growth condition with complete medium.** MCF7 parental, LTED, and TAMR cells were cultivated in complete medium, lysed in RIPA buffer, followed by 30 minutes of DNase/Benzonase treatment on ice. Protein clean-up and digestion was performed using manual SP3 with 500 µg protein input. Peptides were enriched for phosphorylation and both the full and phosphoproteome fractions were analyzed by LC-MS/MS for 120 minutes in DIA mode. Peptides and proteins were identified and quantified using Spectronaut (v17) in directDIA+ mode. Phosphorylated peptides were collapsed to the site-level using the Perseus plug-in PeptideCollapse with a localization cut-off at 0.95. (n=3)

**Suppl. Data 3: Total proteome statistics of MCF7 long-term estrogen deprived (LTED) and tamoxifen resistant (TAMR) vs. parental cells, respectively.** Statistical analysis was performed using the eBayes function of the limma R package and results are presented for LTED vs. parental (Sheet 1) and for the TAMR vs. parental (Sheet 2) comparisons. P-values were adjusted using the Benjamini-Hochberg method.

**Suppl. Data 4: Reactome pathway overrepresentation analysis of total proteomes measured in MCF7 parental, long-term estrogen deprived (LTED) and tamoxifen resistant (TAMR) cells.** Reactome pathway overrepresentation analysis was performed, using a threshold of fold-change > 2 (UP) or < 0.5 (DOWN) and an adjusted p-value < 0.05. Shown are overrepresented Reactome pathways in comparisions LTED vs. parental and TAMR vs. parental, each with direction (up or down) in the respective comparison.

**Suppl. Data 5: Phosphoproteome statistics of MCF7 long-term estrogen deprived (LTED) and tamoxifen resistant (TAMR) vs. parental cells, respectively.** Statistical comparison was performed on the identified, localized and quantified phosphosites using the eBayes function of the limma R package. P-values were adjusted using Benjamini-Hochberg. Results are presented in Sheet 1 for LTED vs. parental and in Sheet 2 for the TAMR vs. parental comparisons.

**Suppl. Data 6: Kinase activities of MCF7 long-term estrogen deprived (LTED) and tamoxifen resistant (TAMR) vs. parental cells, respectively.** Kinase activities were calculated based on the t-statistics, using decoupleR and Omnipath. Results are presented for LTED vs. parental (Sheet 1) and for the TAMR vs. parental (Sheet 2) comparisons.

**Suppl. Data 7: Total proteome result of EGF-stimulated MCF7 parental, long-term estrogen deprived (LTED) and tamoxifen resistant (TAMR) cells.** MCF7 parental, LTED, and TAMR cells were serum-starved for 24 hours to deplete growth factors, followed by stimulation with 5 nM EGF for the indicated duration (in minutes), where “C” represents cells without EGF stimulation (n=3). Protein clean-up and digestion was performed using SP3 with 500 µg protein input. Peptides were enriched for phosphorylation and both the full and phosphoproteome fractions, were analyzed by DIA LC-MS/MS. Proteins were identified using Spectronaut (v17).

**Suppl. Data 8: Phosphoproteome result of EGF-stimulated MCF7 parental, long-term estrogen deprived (LTED) and tamoxifen resistant (TAMR) cells.** MCF7 parental, LTED, and tamoxifen-resistant (TAMR) cells were serum-starved for 24 hours to deplete growth factors, followed by stimulation with 5 nM EGF for the indicated duration, where “C” represents cells without EGF stimulation (n=3). Protein clean-up and digestion was performed using SP2 with 500 µg protein input. Peptides were enriched for phosphorylation and both the full and phosphoproteome fractions were analyzed by DIA LC-MS/MS. Proteins were identified using Spectronaut (v17) and the localization cut-off was set to 0.95 for phosphosite identification with the PeptideCollapse plugin in Perseus.

**Suppl. Data 9: Kinase activities of EGF stimulated MCF7 parental, long-term estrogen deprived (LTED) and tamoxifen resistant (TAMR) cells compared to unstimulated cells.** Statistical analysis of phosphosite intensities compared to the control (“C”, i.e., without EGF stimulation) was performed for the different stimulation-times (in minutes) using the eBayes function of limma (n = 3). Kinase activities were calculated on the t-statistics of the respective comparisons, using decoupleR and Omnipath.

**Suppl. Data 10: Kinase activities of EGF-stimulated MCF7 long-term estrogen deprived (LTED) and tamoxifen resistant (TAMR) vs. parental cells at each time point.** Statistical analysis of phosphosite intensities compared to the respective matched time point in the parental cells was performed using the eBayes function of limma (n=3). Kinase activities were calculated on the t-statistics of the respective comparisons, using decoupleR and Omnipath.

**Suppl. Data 11: Annotated PAK1 target sites in the Omnipath database.** Annotated PAK1 substrates {Turei, 2021 #20532} were retrieved from the Omnipath database (Accessed: 20250901).

**Suppl. Data 12: Predicted PAK1 target sites.** Predicted PAK1 substrates were obtained from Johnson et al. [50] by filtering for phosphosites with PAK1 in at least the 98^th^ percentile and ranking higher than 5.

**Suppl. Data 13: Total proteome result of PAK1-inhibited MCF7 parental, long-term estrogen deprived (LTED) and tamoxifen resistant (TAMR) cells.** MCF7 parental, LTED, and TAMR cells were treated with 6 µM PAK1 inhibitor NVS-PAK1-1 for 1 hour (n=3). Proteins were prepared for LC-MS/MS analysis by automated SP3 and phospho-enrichment of 55 µg protein as input material. Both the total and phospho proteome fractions were analyzed by LC-MS/MS for 120 minutes in DIA mode. Peptides and proteins were identified and quantified using Spectronaut (v17) in directDIA+ mode. (n=3)

**Suppl. Data 14: Total proteome statistics of PAK1-inhibited MCF7 parental, long-term estrogen deprived (LTED), and tamoxifen resistant (TAMR) vs. respective DMSO-treated control cells.** Statistical comparison of PAK1 inhibitor NVS-PAK1-1 (PAKi) treated vs. DMSO control for parental, LTED, and TAMR cells (data from Suppl. Data 13), respectively, was performed using the eBayes function of limma (v. 3.58.1). P-values were adjusted using Benjamini-Hochberg (n=3). Results are presented for parental vs. DMSO (Sheet 1), for LTED vs. DMSO (Sheet 2), and for TAMR vs. DMSO (Sheet 3) comparisons.

**Suppl. Data 15: Phosphoproteome results of PAK1-inhibitor- and DMSO-treated MCF7 parental, long-term estrogen deprived (LTED) and tamoxifen resistant (TAMR) cells.** MCF7 parental, LTED, and TAMR cells were treated either with 6 µM PAK1 inhibitor NVS-PAK1-1 or DMSO for 1 hour (n=3). Proteins were prepared for LC-MS/MS analysis by automated SP3 and phospho-enrichment was performed with 55 µg protein as input material for every sample. Both the full and phospho proteome fractions were analyzed by LC-MS/MS for 120 minutes in DIA mode. Peptides and proteins were identified and quantified using Spectronaut (v17) in directDIA+ mode. Phosphorylated peptides were collapsed to the site-level using the Perseus plug-in PeptideCollapse with a localization cut-off at 0.95.

**Suppl. Data 16: Phosphoproteome statistics of PAK1-inhibited MCF7 parental, long-term estrogen deprived (LTED), and tamoxifen resistant (TAMR) vs. respective DMSO-treated control cells.** Statistical comparison of PAK1 inhibitor NVS-PAK1-1 (PAKi) treated vs. DMSO control for parental, LTED, and TAMR cells (data from Suppl. Data 15), respectively, was performed using the eBayes function of limma (v. 3.58.1). P-values were adjusted using Benjamini-Hochberg (n=3). Results are presented for parental vs. DMSO (Sheet 1), for LTED vs. DMSO (Sheet 2), and for TAMR vs. DMSO (Sheet 3) comparisons.

**Suppl. Data 17: Kinase activities of PAK1-inhibited MCF7 parental, long-term estrogen deprived (LTED), and tamoxifen resistant (TAMR) vs. respective DMSO-treated control cells.** Kinase activities were calculated for MCF7 parental, LTED, and TAMR cells, respectively, after treatment with PAK1 inhibitor NVS-PAK1-1 (PAKi) or DMSO (data from Suppl. Data 15), based on the t-statistics of the respective comparison, using decoupleR and Omnipath. Kinases and respective Omnipath scores are presented for parental vs. DMSO (Sheet 1), for LTED vs. DMSO (Sheet 2), and for TAMR vs. DMSO (Sheet 3) comparisons.
